# Mammalian Life History: Weaning and Tooth Emergence in a Seasonal World

**DOI:** 10.3390/biology13080612

**Published:** 2024-08-12

**Authors:** B. Holly Smith

**Affiliations:** 1 Museum of Anthropological Archaeology, University of Michigan, Ann Arbor, MI 48109, USA; bhsmith@umich.edu; 2 Center for the Advanced Study of Human Paleobiology, George Washington University, Washington, DC 20052, USA

**Keywords:** life history, Mammalia, Eutheria, primates, weaning, gestation length, tooth eruption, parent-offspring conflict, altricial, precocial, brain weight

## Abstract

**Simple Summary:**

Mammals nurse their young through rapid early growth. Most placental mammals bridge the period with a set of small, temporary ‘deciduous’ or ‘milk’ teeth. At some point, a mother weans her young, who must then feed independently to survive. How tooth eruption integrates with gestation, birth and weaning is explored here for 71 species in nine mammalian orders. Body weights range from 22 g to 4300 kg and maternal investment (gestation plus nursing) ranges from 6 weeks to more than 7 years. These mammals differ widely at birth, from no teeth to all deciduous teeth emerging, but commonalities appear when infants transit to independent feeding. Weaning takes place with an entire deciduous dentition, closest in time to emergence of the first permanent molars and well before second molars emerge. Adult body size explains less about tooth eruption than expected. Instead, many mammals, from monkey to moose, limit maternal investment (from initial pregnancy to young with first molars) to just under one year, timing infant development to annual cycles. Mammals that invest multiple years in their young include several critically endangered species. Integrating tooth emergence into life history gives insight into living mammals and builds a framework for interpreting the fossil record.

**Abstract:**

The young of toothed mammals must have teeth to reach feeding independence. How tooth eruption integrates with gestation, birth and weaning is examined in a life-history perspective for 71 species of placental mammals. Questions developed from high-quality primate data are then addressed in the total sample. Rather than correlation, comparisons focus on equivalence, sequence, the relation to absolutes (six months, one year), the distribution of error and adaptive extremes. These mammals differ widely at birth, from no teeth to all deciduous teeth emerging, but commonalities appear when infants transit to independent feeding. Weaning follows completion of the deciduous dentition, closest in time to emergence of the first permanent molars and well before second molars emerge. Another layer of meaning appears when developmental age is counted from conception because the total time to produce young feeding independently comes up against seasonal boundaries that are costly to cross for reproductive fitness. Mammals of a vast range of sizes and taxa, from squirrel monkey to moose, hold conception-to-first molars in just under one year. Integrating tooth emergence into life history gives insight into living mammals and builds a framework for interpreting the fossil record.

## 1. Introduction

What makes a mammal? Most would answer that mammals nurse their young and are covered in hair. Hair, of course, insulates active endothermic bodies, while nursing provides immune factors and nutritious, digestible food that supports rapid growth while the gut and dentition mature, allowing young to be weaned onto an adult diet [1,2,3]. If the adult diet has complexities, the close association between mother and young while nursing facilitates learning what to eat and how to procure it [4,5]. Feeding independence requires a dentition and gut that can process available food, just as it requires a workable locomotor system to forage independently.

Placental mammals bridge early rapid growth with a set of small, temporary, ‘deciduous’ or ‘milk’ teeth. A period of a milk-only diet is often followed by mixed feeding that progresses toward dominance of an adult diet, a transition (or ‘process’) that can occupy days, months or years [6], but eventually young are weaned from milk. Nursing can adapt to circumstance, shortening when young are flourishing or extending in hard times [7]. Prolonged supplemental nursing benefits the young, but also increases the time needed for a mother to recoup her energy and reproduce again, making weaning the classic example of parent–offspring conflict [8,9]. In placental mammals living today, a single month of nursing is common; more than a year is rare (see below).

Mammals also live in a changing world in which species of all sizes must fit their lives into the same diuranlht, lunar and annual cycles. Because the axis of the earth is tilted, solar radiation that reaches the Earth differs by latitude and changes day by day as the Earth orbits the sun. Day length varies least at the equator, but because wind and rain are also affected by sunlight, climate seasonality is global [10], affecting plant communities and all life that feeds on them. How elements of reproduction (estrus, mating, gestation, birth and weaning) are tuned to lunar and annual cycles is increasingly well documented [10,11]. Somatic growth and development are also tuned to daily and higher-level cycles. Photoperiod, for example, is a cue for hormones affecting mammalian growth [12]. Rhythms of growth observed in bone and the dental hard tissues—enamel, dentin and cementum—document daily and higher-order cycles of cellular growth [13,14,15,16].

Some specialized mammals have lost all of their teeth (particularly when predators are far larger than their prey, as for baleen whales and various ant-eaters), but enough mammals retain teeth and a generalized system of tooth replacement to make it worth searching for commonalities in weaning relative to eruption of the dentition, a transition when life depends on the functional success of occlusion and mastication [17,18,19,20]. 

A previous study showed that the emergence of the mandibular first permanent molar (M_1_) is highly correlated with a series of life-history traits in primates [17]. For these well-studied mammals, M_1_ emergence appeared to be nearly equivalent to weaning [21,22,23], although the relationship has been questioned and exceptions noted [24,25]. The present study reconsiders the relationship of tooth emergence to weaning by first examining the fine-grained and high-quality data for primates [26,27] to generate hypotheses, and second by testing hypotheses against all data for placental mammals, observing commonalities and noting when exceptions are revealing. The results increase our understanding of growth and development of living mammals and should lead to more dynamic inferences about the life history of extinct species, because estimates for age of tooth emergence are often recoverable in the fossil record [23,28,29,30,31,32,33,34]. 

## 2. Background

Gestation and lactation are often about equal in duration in placental mammals living today, although lactation remains far more dominant in marsupials [35]. Gestation may be the more efficient means of energy transfer [36], but lactation has added behavioral components that allow mothers flexibility in responding to environmental conditions and choices that impact future reproduction. ‘Flexible’ does not mean cheap: the daily energy cost of lactation is even higher than that of pregnancy, draining body fat and bone minerals, and delaying return to estrus and ovulation [2,35,37,38]. Returning to a positive energy balance has been shown to be critical to the return of ovulation in humans [39,40], and for female mammals in general, energy availability and hormonal activity are said to interact on a ‘minute-to-minute basis’ [41] (p. 235). For free-living mammals, cases where lactation and subsequent pregnancy overlap are always notable [42,43,44,45,46,47] because the mother is ‘metabolizing for three’—herself, her pregnancy and a nursing infant [40] (p. 559). 

Gestation length and age of infant weaning are two of the best measures of a species’ position on the fast–slow continuum of life history [48]. Many mammals add even a third phase of investment in their young. Young carnivores may be weaned and able to process adult food, but still depend on a mother who pulls down kills [49]. Food sharing by offering, soliciting and theft is well known in social primates—quintessentially humans—and is also documented in a variety of social carnivores and rodents [4,50,51,52]. Similarly, episodic nursing of an infant by another mother is known in social mammals, although it may be costly [53]. This third phase of indirect investment, even if we could quantify it, would not be included here because costs incurred by sharing food or protecting young may be distributed across kin or group members [54,55,56,57,58]. 

**Figure 1:**
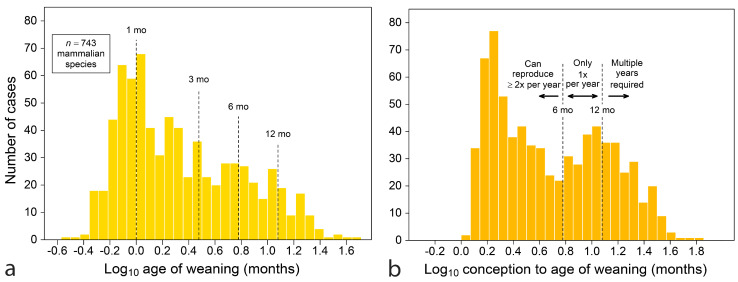
Distribution of life-history characteristics for 743 nonvolant placental mammals: (**a**) age of weaning (duration of nursing); and (**b**) conception to age of weaning (gestation length plus weaning age) or total time to produce young that feed independently. Peaks recognizable in (**a**) take on new meaning in (**b**), where a trough between modes separates species that can reproduce more than once a year from those that cannot (barring concurrent pregnancy and nursing). Data from Ernest [59].

A large compendium of life-history data [59] allows sweeping views of maternal adaptations. Figure 1a displays a histogram of the age of weaning for 743 nonvolant placental (NVP) mammals, about 20% of species recognized at the time it was assembled [60]. Nursing is commonly short in duration, with a mode near one month. Even with the inclusion of some large marine mammals, few species nurse beyond 12 months.

A deeper adaptation to seasonality is revealed by counting the age of weaning from conception (adding gestation length to weaning age), which produces a variable that represents the time a female invests in one reproductive event, from initiating pregnancy to producing young that feed independently (Figure 1b). For the same 743 species, the new histogram is bimodal, with the highest mode at about two months and the second at about eleven months, with a distinct trough between the two. The location of the trough near six months argues that annual cycles have shaped adaptations because it coincides with a limit beyond which a species cannot reproduce more than once within an annual cycle. 

A simple interpretation of Figure 1b is that there are at least three reproductive strategies represented: (1) reproduce twice a year or more, (2) reproduce only once a year and (3) take multiple years to raise young to independent feeding. While no such data-rich demonstration can be made about teeth, it is worth looking for evidence of lunar, semi-annual and annual timing in teeth, because weaning and tooth emergence are two aspects of raising young to feeding independence. 

## 3. Materials and Methods

*Dental notation*: Abbreviations for teeth follow Smith et al. [26], where capital letters indicate the permanent teeth (I, incisors; C, canines; P, premolars; M, molars) and lower-case letters preceded by “d” denote deciduous teeth (di, dc, dp). Numeric superscripts and subscripts mark tooth position in series and by jaw, upper or lower (M^1^, M_1_) and ‘M1’ refers to all four first permanent molars.

*Choice of teeth for study*: Generalized placental mammals are diphyodont, with two tooth generations for anterior teeth and a single generation of three posterior molars. The present study considers events that are widely comparable across species: the age of completion of the entire deciduous dentition (emergence of the final deciduous tooth at any locus, over both jaws when data are available) along with ages of M_1_ and M_2_ emergence. This series, erupting largely front to back, is tuned to facial growth. Ontogenetically, permanent molars are extensions of the deciduous dentition, derived from an extending dental lamina, whereas replacement teeth I1-P4 bud off epithelial connections from their deciduous predecessors [61,62]. Variations in eruption sequence across placentals can be attributed almost entirely to the timing of replacement teeth [63], which have diversified to erupt in widely different sequences relative to permanent molars, from following M3 (early Cenozoic mammals, tree shrews and smaller artiodactyls) [63,64,65,66] to preceding M1 (incisors of many carnivores) [67,68,69] and even to erupting prenatally (northern fur seal) [70]. The interaction of growth rate with birth status and deciduous tooth wear likely influences the evolution of mammalian tooth replacement; see also [71,72].

*Age of tooth emergence*: Data on tooth emergence were gathered from original sources, including literature from wildlife conservation, mammalogy, zoological gardens and medical research. Because most studies observed live animals, ‘emergence’ was usually defined as the first appearance of a cusp cut through the gum (tooth ‘eruption’ is the process). Data for the mandible are more often available than for the maxilla and for earlier versus later erupting teeth. Subjects of study included free-living and captive animals and data could be cross-sectional, longitudinal or mixed. Domestic species were omitted because the maturation of domesticated animals versus their wild relatives is too large a subject to be undertaken here. 

The original sources presented data in many formats. Criteria for inclusion were that the ages of individual subjects were known or very closely estimated, gaps between observations were reasonable and emergence of teeth was reported in a manner that could be made comparable to other data. The aim was to capture the age at which 50% of subjects have the tooth erupted. Point estimates were found by cumulative functions such as probit analysis [73], or by taking the midpoint in age between the last in series of unerupted teeth to the age of the first in a series of erupted teeth [74], or by correcting for exam interval in longitudinal data [75]. Thus, the precise ages reported here may not appear in the original studies. 

For the tree shrew, the data approach the ideal, where infants were looked at daily through to completion of the deciduous dentition [76]. Other reports were omitted for a time gap around M_1_ [77] or emergence gauged relative to the alveolus [78], but considerable effort was made to interpret the sparse and difficult available records for several large slow-growing mammals—the orangutan, rhinoceros, tapir, giraffe, hippopotamus, hyena and panda—all of which are inadequately described. Original studies of these mammals often mixed observations of young live animals with extrapolations made to older de-fleshed specimens, although the tapir M_1_ datum is based only on museum specimens (see table notes). Despite the underlying variability of approaches in original sources, the ages of M_1_ emergence presented here differ by an average of only 0.10 ± 0.07 year from those of Veitschegger and Sánchez-Villagra [71] for the 15 species that overlap. The difference is proportionately large only for *Muntiacus reevesi*, the Muntjac deer (0.207 vs. 0.019 yr). 

All ages are tabulated in years. Although months are useful for modeling seasonality, they are poor units for scientific computation.

*Birth status and the deciduous dentition*: Studying deciduous tooth emergence adds a complication because mammals range widely in dental development at birth—one indicator of position on the spectrum of altricial (less developed) to precocial (well developed) birth status [79]. Newborn mammals vary in degree of development across organ systems, but are commonly ranked by hair, ears, eyes, teeth, thermoregulation and movement [80,81,82]. Tables (below) report the number of deciduous teeth already cut through the gum at birth along with birth status as a general category. In addition to “altricial” and “precocial”, two categories give nuance to largely precocial young, where ‘*Tragerjunge*’ (transported) are young that must be carried and ‘*Lagerjunge*’ are those held back in a nest for a time [6]. Teeth already emerged at birth drop out of further analysis because emergence day in utero is rarely known (but see [70,83]). 

For most of these mammals, the last deciduous tooth to emerge is dp^4^, although the deciduous canine may lag. Completion was tallied without regard to the first premolar, a tooth position lost in many lineages. Although counted in the permanent dental formula, the first premolar is commonly unreplaced and embryologically best ascribed to the deciduous dentition, although a few species are known to replace a dp1 [84]. When unreplaced, dp1 can emerge very late, sometimes in tandem with M_1_ [63]. The tooth is not present in living primates but can be seen in some extinct taxa [85]. 

Most of the mammals reviewed here have a functioning deciduous dentition, but several of the carnivores (*Enhydra*, *Mirounga* and *Mephitis*) suppress many or all to a vestige resorbed or shed in utero [86]. In primates, *Tarsius* [87] and *Propithecus* [87,88,89] each suppress a few deciduous teeth. Rodents may retain one or more deciduous premolars, as in *Sciurus*, or show no deciduous teeth, as in *Myodes*.

*Primates*: Table 1 compiles ages of tooth emergence and other aspects of life history for 22 species in ten families of living primates, adding two extinct species for which age of emergence of M_1_, body weight and brain weight have all been reconstructed. One genus, *Macaca*, appears twice to give an example of intrageneric similarity. Note that female body weights are used for primates, whereas comparisons across all 71 placental species use an estimate of overall species weight (where sex-specific weights are not always available). 

*Non-primate mammals*: Table 2 compiles the ages of tooth emergence and other aspects of life history for 47 species across 26 families representing eight orders of placental mammals, expanding on limited data published previously [63]. The domestic Camelidae are not included. Elephants are included; although their teeth move forward to be shed throughout life, the teeth are homologous in number and identity with those of other placentals [90]. Modern cetaceans are not included because tooth replacement and tooth homology are lost. Marsupials are not included because they differ in the way nursing and tooth eruption bridge growth [84,91,92]. All species in Table 2 have M_1_, but several carnivore species have eliminated M_2–3_ (marked ‘x’ in Table 2). 

**Table 1:** Tooth emergence and other life history parameters for 22 living and two extinct primate species, with female body weight plus overall weight ^1^.

	Birth Status		Weight			Timed Events in Life History (Years)				
Primates Family *Genus Species*	Category ^2^	No. Decid. Birth	♀ Body (kg)	Body (kg)	Brain (g)	Gest. Length	Weaning	Complete Deciduous Dentition	M_1_ Emergence	M_2_ Emergence
Cheirogaleidae										
*Cheirogaleus medius* [93]	Tr	12	0.182	0.197	2.60	0.169	0.167	0.030	0.070	0.10
Lemuridae										
*Lemur catta*	Tr	7	2.205	1.960	22.90	0.370	0.290	0.115	0.340	0.57
*Eulemur fulvus*	Tr	2	1.775	2.292	25.77	0.323	0.370	0.212	0.420	0.61
*Varecia variegata*	Tr	2	3.700	3.575	32.12	0.269	0.300	0.213	0.480	0.69
Indriidae										
*Propithecus verreauxi*	Tr	16 + V	3.150	2.955	26.21	0.406	0.490	prenatal	0.220	-
Galagidae										
*Galago senegalensis* [94]	Tr	12	0.199	0.213	3.96	0.345	0.233	0.030	0.100	-
Tarsiidae										
*Tarsius bancanus* [87,95]	Tr	16 + V	0.114	0.126	3.16	0.488	0.220	0.011	0.037	-
Callitrichidae										
*Callithrix jacchus*	Tr	0	0.322	0.320	7.24	0.395	0.167	0.104	0.310	0.59
*Saguinas fuscicollis* [96]	Tr	?	0.320	0.401	7.94	0.403	0.250	0.125	0.380	0.58
Cebidae										
*Cebus apella*	Tr	8	2.350	2.936	66.63	0.425	0.740	0.393	1.100	2.17
*Saimiri sciureus*	Tr	2	0.950	0.852	24.14	0.466	0.480	0.118	0.370	0.59
*Aotus trivirgatus*	Tr	0	0.736	0.989	16.85	0.389	0.210	0.108	0.360	0.53
Cercopithecidae										
*Macaca mulatta*	Tr	0	5.340	6.793	88.98	0.460	0.803	0.437	1.350	3.50
*Macaca nemestrina*	Tr	0	5.940	8.821	105.59	0.469	1.000	0.410	1.370	3.34
*Papio cynocephalus*	Tr	0	11.900	17.150	163.19	0.488	0.993	0.596	1.665	3.79
*Chlorocebus aethiops*	Tr	0	3.200	3.720	65.00	0.447	0.710	-	0.830	1.83
*Trachypithecus vetulus*	Tr	0	6.525	6.237	61.29	0.556	0.625	0.203	-	-
Hylobatidae										
*Hylobates agilis* [94,97]	Tr	2	5.82	5.850	91.16	0.562	2.000	0.450	1.36	2.68
Hominidae										
*Pongo pygmaeus* ^3^ [98]	Tr	0	35.80	57.150	377.38	0.682	6.500	1.160	≅3.5	≅6
*Gorilla gorilla*	Tr	0	71.50	120.950	490.41	0.701	3.800	0.994	3.50	6.58
*Pan troglodytes* [99,100]	Tr	0	39.70	44.967	368.35	0.658	4.710	1.120	3.33	6.45
*Homo sapiens* [101,102]	Tr	0	42.00	49.500	1290.00	0.767	2.800	2.333	5.75	10.73
*Australopithecus africanus* [100,103,104]		-	30.20	35.500	433.95	-	-	-	3.20	-
Megaladapidae										
*Megaladapis edwardsi* ^4^ [33]	-	-	88.00		137.00	>0.54	-	-	0.92	>1.39

^1^ Sources: Dental data [26], female body weight [27], body and brain weight [105,106,107,108,109,110], gestation and weaning [27,59], except as noted. ^2^ Birth status: Dietrich Starck categories of newborn development in Langer [6] (A, altricial; P, precocial; Tr, *Tragejunge*; Lj, *Lagerjunge*); number of teeth emerged at birth (0, ‘naked gums;’ ’16 + V’ = 16 functional teeth plus other vestigial teeth). ^3^ *Pongo pygmaeus* life history taken from Borneo, but dental data mix Borneo and Sumatra. The final deciduous tooth was observed emerging in 7 mother-reared infants from diverse zoos; permanent molars based on one Sumatran male born almost 100 years ago at the Dresden Zoo, although data points from a dozen other captive juveniles do not conflict [111]. For *Pongo* a regression predicts M_1_ emergence at 3.37 yr (n = 17, R^2^ = 0.99) and M_2_ at 7.23 yr (n = 15, R^2^ = 0.98) for a deciduous dentition complete at 1.16 years. ^4^ *Megaladapis* is not known to be sexually dimorphic and 88 kg is a midrange weight.

**Table 2:** Tooth emergence and other life history parameters for 47 non-primate species of placental mammals ^1^.

	Birth Status		Weight		Timed Events in Life History (Years)				
Order Family *Genus Species*	Category ^2^	No. Decid. Birth	Body (kg)	Brain (g)	Gest. Length	Weaning	Complete Deciduous Dentition	M_1_ Emergence	M_2_ Emergence
**Scandentia**									
Tupaiidae									
*Tupaia glis* ^3^ [76]	A	0	0.170	3.20	0.123	0.099	0.066	≅0.085	-
**Rodentia**									
Cricetidae									
*Myodes glareolus* [112]	A	0	0.022	0.52	0.058	0.060	-	0.027	0.041
Hystricidae									
*Hystrix africaeaustralis* [113]	Lj	4	17.667	22.42	0.256	0.277	0.038	0.188	0.458
Sciuridae									
*Sciurus carolinensis* [114]	A	0	0.503	7.41	0.121	0.178	-	0.134	-
*Tamiasciurus hudsonicus* [115]	A		0.202	3.67	0.093	0.137	-	0.128	-
Thryonomyidae									
*Thryonomys swinderianus* [116]	P	4	3.580	13.28	0.425	0.083	-	0.058	0.334
**Perissodactyla**									
Equidae									
*Equus burchelli* [117]	P	0	227.600	612.00	1.00	0.750	0.420	0.875	1.750
Tapiridae									
*Tapirus terrestris* ^4^ [118]	P	0	158.489	181.97	1.09	0.792	-	≈1.2	-
Rhinocerotidae									
*Ceratotherium simum* [119,120]	P	0	1396.000	642.00	1.315	1.00	0.384	2.75	-
*Diceros bicornis* [120,121]	P	0	954.993	616.60	1.299	1.66	0.384	2.0	4.0
**Artiodactyla**									
Antilocapridae									
*Antilocapra americana* [122]	P	≥4	46.750	125.60	0.690	0.250	0.058	0.167	0.458
Bovidae									
*Aepyceros melampus* [123]	P	8	47.000	181.70	0.559	0.460	0.125	0.330	0.792
*Antidorcas marsupialis* [124]	P	20	36.518	135.00	0.468	0.329	-	0.083	0.625
*Bison bonasus* [125,126]	P	≥18	900.000	665.142	0.740	0.550	0.104	0.542	1.417
*Connochaetes taurinus* [127]	P	6	154.667	365.60	0.690	0.667	-	0.458	1.167
*Hemitragus jemlahicus* [128]	P	≅14	70.000	166.00	0.575	0.414	0.019	0.208	0.958
*Saiga tatarica* [129]	P	8	40.000	96.67	0.416	0.243	-	0.125	0.292
*Sylvicapra grimmia* [130,131]	P	8	12.625	77.20	0.452	0.287	0.043	0.229	0.790
*Taurotragus oryx* [132,133]	P	20	531.50	578.00	0.729	0.500	prenatal	0.528	1.50
Cervidae									
*Alces alces* [134]	P	≥8	272.160	406.50	0.666	0.274	0.067	0.231	0.666
*Cervus elaphus* [135,136]	P	8	120.177	379.20	0.671	0.333	0.019	0.330	1.125
*Muntiacus reevesi* [137]	P	22	13.500	70.17	0.545	0.166	prenatal	0.207	0.577
*Odocoileus virginianus* [138]	P	≥8	65.000	210.00	0.559	0.330	0.058	0.180	0.656
*Rangifer tarandus* [139]	P	≅22	106.360	299.00	0.625	0.330	prenatal?	0.170	0.833
*Giraffa camelopardalis* [140]	P	18	828.000	678.30	1.252	1.0	0.040	0.917	2.0
Hippopotamidae									
*Hippopotamus amphibius* [141]	P	6+	1553.000	651.00	0.641	0.934	0.75	≈1.5	8?
Suidae									
*Potamochoerus porcus* [142]	Lj	8	130.000	155.60	0.334	0.250	0.265	0.438	1.212
*Sus scrofa* [143]	Lj	8	117.000	188.20	0.315	0.290	0.261	0.472	1.055
Tayassuidae									
*Pecari tajacu* [144]	Lj	6	8.430	66.00	0.397	0.130	0.165	0.400	0.819
Tragulidae									
*Hyemoschus aquaticus* [42]	P	-	10.800	25.20	0.479	0.493	-	0.417	0.830
**Carnivora**									
Canidae									
*Canis mesomelas* [67]	A	0	8.500	51.42	0.173	0.164	0.053	0.356	0.389
*Cerdocyon thous* [145]	A	0	5.240	41.80	0.151	0.250	0.089	-	-
*Vulpes vulpes* [68]	A	0	8.000	43.38	0.143	0.131	0.079	0.327	0.356
Felidae									
*Lynx rufus* [146]	A	0	8.900	57.97	0.178	0.238	0.135	0.442	x ^5^
*Panthera leo* [69]	A	0	161.500	223.63	0.299	0.602	0.208	1.042	x
Hyaenidae									
*Crocuta crocuta* [147,148,149]	Lj	16	63.000	144.03	0.301	0.726	0.164	0.833	x
Mustelidae									
*Eira barbera* [150]	A	0	3.910	35.87	0.181	0.226	0.242	0.533	0.568
*Enhydra lutris* [151]	?	10 + V	23.500	125.21	0.384	0.481	0.060	0.580	-
*Mephitis mephitis* [152]	A	V	2.090	10.28	0.173	0.164	prenatal	0.134	0.138
*Neovison vison* [153]	A	0	0.645	7.00	0.115	0.133	0.110	0.157	0.171
Procyonidae									
*Procyon lotor* [154]	A	0	5.530	40.04	0.173	0.246	0.135	0.214	-
Ursidae									
*Ailuropoda melanoleuca* [155]	A	0	108.400	235.10	0.369	0.885	0.384	1.0	-
*Ursus arctos* ^6^ [156]	A	0	172.720	338.30	0.282	0.584	0.315	0.372	0.567
**Pinnepedia** (sub-order)									
Phocidae									
*Mirounga leonina* [157]	P	V	2470.625	700.88	0.603	0.058	prenatal	0.074	x
**Hyracoidea**									
Procaviidae									
*Procavia capensis* [158]	P		3.090	20.07	0.589	0.317	-	0.542	1.167
**Probosciodea** Elephantidae									
*Loxodonta africana* [159,160]	P	4	4353.167	5806.67	1.836	3.500	-	6.0	14.50
**Macroscelidea**									
Macroscelididae									
*Macroscelides proboscideus* [108,161]	P		0.039	1.34	0.178	0.052	-	0.095	0.233

^1^ Sources: dental data in table; body and brain weights [105,106,107,108,109], gestation and weaning [59,106,107], except as noted. ^2^ Birth status: Dietrich Starck categories of newborn development in Langer [6] (A, altricial; P, precocial; Tr, *Tragejunge*; Lj, *Lagerjunge*); number of teeth emerged at birth (0, ‘naked gums’; ‘10 + V’, 10 functional teeth plus other vestigial teeth); the sea otter, *Enhydra lutris*, is unique, born in the sea with eyes open and hair through. ^3^ For *Tupaia*, M_1_ was previously predicted [63] to emerge 5 days after its complete deciduous dentition by a primate regression (R^2^ = 0.99), the only predicted datum here. ^4^ For *Tapirus*, adding Simpson’s record of a 13-month-old with no M_2_ erupted (implying that M_1_ had erupted) to British Museum Natural History specimen 75–571, a tagged 468-day-old zoo specimen with M_1_s erupting, not quite emerged, makes an age category with 50% M_1_ emerged at 1.1–1.3 yr. Published deciduous completion data are contradictory. ^5^ x, not present in species. ^6^ For cubs overwintered, *Ursus arctos* weaning estimated at 1.15 yr; for overwintering in *Cervus elaphus*, see text.

*Other life history parameters*: Body weight, brain weight, gestation length and age of weaning are taken from literature and online sources (see table notes). The smallest mammal included is the meadow vole at 22 g; the largest is the African elephant at over 4000 kg. The use of three decimal points reflects the need to span this great range.

Some species included here employ delayed implantation (e.g., *Ursus*, *Ailuropoda* and *Enhydra*) or diapause (*Callithrix*). By convention, this time is usually included in gestation length, but reports of duration of such pregnancies consequently vary.

‘Weaning’ is usually considered to be an estimate of the age of cessation of nursing in free-living animals, although individual field workers and authors may differ. Like all data from compendia, these are estimates of typical or average values. Humans are represented here by data for extractive foragers where weaning, at 2.8 years, is the latest value that could be assigned to typify a human economy [162,163]. Other values for *Homo sapiens* are taken from indigenous Australasia before 1970, where body weights were light [101] and tooth emergence early [102]; brain weight is from Oceania [164].

Two large Holarctic mammals, *Cervus elaphus* (red deer/elk/wapiti) and *Ursus arctos* (brown bear), may or may not nurse present offspring into the following winter—for *C. elaphus*, nursing even while pregnant [165,166,167]. Pre-winter weaning appears in Table 2, as of most relevance to the dentition, but over-wintering is discussed below.

The two greatest ages of weaning documented in mammals belong to orangutans, with 6.5 years reported for Bornean *Pongo pygmaeus* [98] and 8.5 years for Sumatran *P. abelii* [168]. Because extremely long maternal investments grade toward extinction, the range 6.5–8.5 is graphed as ‘maximum.’ Indeed, *P. abelii* is highly likely to be extinct on a time horizon of a few hundred years, whereas *P. pygmaeus* survival for the next 1000 years appears more likely [169].

*Counting age*: In graphs, age is counted from birth or from conception (adding gestation length to age since birth). For events early in life, counting age from conception helps make sense of comparisons between altricial and precocial young, where time in utero differs greatly. It also expresses age as total time of direct maternal investment (as in Figure 1b) and puts numerical error in developmental perspective. For example, the tarsier, after a gestation of some 178 days, apparently completes its deciduous dentition sometime between birth and day eight [87,95]. If we settle on day four as a point estimate, we may easily err by four days. Is this an error of 100% (4/4) or 2% (4/182)? It all depends on how one counts age. Counting age from conception matters less for later events because gestation length diminishes as a proportion of age.

*Analysis*: Initial analyses consider primates separately because these data are high in overall quality [26], available across the order and include extinct species. Female body weights, preferable for analyses concerning gestation and lactation, are more commonly available. Moreover, the hypotheses discussed here were developed while studying primates [17].

Raw data are transformed to logarithms, as is standard practice, because growth and development are proportional processes. Because age of weaning and age of emergence of M1 are two measures of the time required to raise young to independent feeding, they are necessarily highly correlated in a log–log ‘mouse to elephant’ comparison. The degree of correlation and level of statistical significance are not primary issues and the transformation of data based on inferred cladistic relationships (e.g., [170]) would obscure the present aim. Instead, the focus is on equivalence, sequence, the relationship to seasonal divisions (particularly six and twelve months), the distribution of error and adaptive extremes.

Results are presented in paired scatter graphs of two kinds: pairs where the y variate is the same and the x variate differs and pairs where graphs are the same, but time is counted from birth and again from conception. 

Findings are considered in an adaptive context because evolution—by selection or by drift—is fast [171,172] and because successful weaning underlies reproductive fitness, see [173].

## 4. Results

### 4.1. Primates

#### 4.1.1. The Dentition at Birth

Newborn primates are precocial in terms of ‘hair through’ and ‘ears and eyes not sealed,’ but motor ability is poorly developed and infants must be transported (Tr for ‘*Tragerjunge*’ in Table 1) [6]. In terms of teeth emerged at birth, primates vary from the dentally precocious *Tarsius* and *Propithecus*, born with most or all deciduous teeth in place, to the great apes and humans, where newborns are toothless for weeks or months [26]. Within anthropoids, platyrrhines are more precocial in tooth emergence at birth than catarrhines (although *Saguinas* probably has fewer teeth at birth [96] than previously reported [26]). No primate has been observed with M1 emerged at birth see also [174,175].

#### 4.1.2. Maternal Investment

Most primates in Table 1 occupy the category of “reproduce once a year” or “take multiple years to raise young” (as in Figure 1b). Only *Cheirogaleus medius*, the fat-tailed dwarf lemur, invests less than one half year in young, but it, like the mouse lemurs [27], is restricted to breeding in a half year, going into torpor in the dry season. The African *Galago senegalensis* invests just over 0.5 years, but its interbirth interval remains at one year, with females spending months in a non-breeding state. In Asia, even the smallest primates invest far more than 0.5 years in each infant, where the 114 g *Tarsius bancanus* female invests 0.70 yrs. Although information about teeth is not available for *Loris tardigradus*, a 132 g female invests 0.93 yrs per infant, more time than callitrichids three times her size (compare in Table 1).

Reproducing once *or* twice per year probably does occur in the Callitrichidae, especially in some *Saguinas* sp. [27]. Other platyrrhine primates in Table 1 appear both above and below a one-year investment. All catarrhines probably invest more than one year in each offspring.

#### 4.1.3. Mapping Weaning onto Tooth Emergence

**Figure 2:**
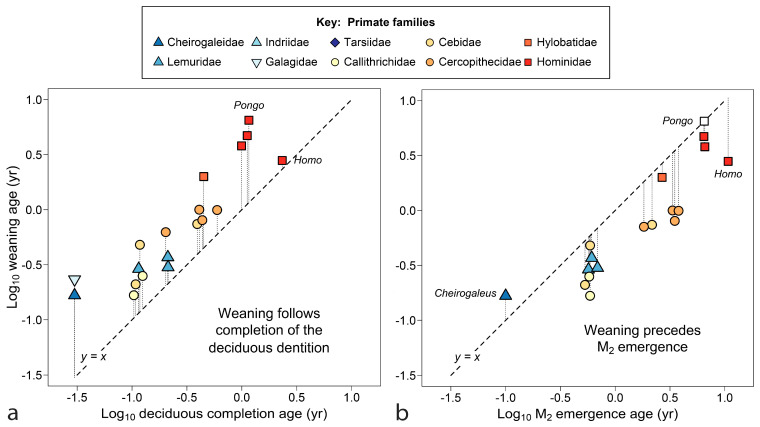
Age of weaning versus age of completion of the deciduous dentition (**a**) and age of M_2_ emergence (**b**) with age counted from birth in both. Residuals (in *y*-direction from the dashed line *y* = *x*) shown for n = 19 primate species as a fine line. Weaning occurs after young have a complete deciduous dentition, but typically well before M_2_ is in place. For *Pongo*, tooth emergence datum is precise at left (**a**), but only approximated at right (**b**) with an unfilled symbol. *Tarsiidae* and *Indriidae* omitted in (**a**) for scale, although both wean infants after deciduous teeth are emerged (their symbols remain in key).

Comparisons in Figure 2 begin to narrow the timing of weaning in relation to tooth emergence for primates. Figure 2a shows that weaning typically occurs after—even substantially after—completion of the deciduous dentition (gingival emergence of all deciduous teeth). All the apes, both lesser (*Hylobates*) and great (*Pan*, *Gorilla* and *Pongo*), show late and latest weaning versus deciduous completion. The extreme position of the orangutan (*Pongo pygmaeus*) in Figure 2a is especially notable because completion of the deciduous dentition is the highest quality data point for tooth emergence in the genus (see Table 1 note). Even human extractive foragers tend to wean after all deciduous teeth have emerged, although with an age of weaning absolutely lower than those of the great apes.

In Figure 2b, most primates are seen to wean offspring well before M_2_ appears, a tooth that appears later in time and farther back in the mouth. The data cluster, but not always along taxonomic lines. The only primate shown here to prolong nursing after the appearance of M_2_ is the precocious lesser dwarf lemur, *Cheirogaleus medius*, where M_2_ emerges at about 5 weeks of age. The Bornean orangutan, *Pongo pygmaeus*, weaned ca. 6.5 yrs, nears equivalence with M_2_ if the tooth erupts at an age comparable to other great apes (ca 6.5 yrs); Sumatran *P. abelii* may break the anthropoid pattern completely.

**Figure 3:**
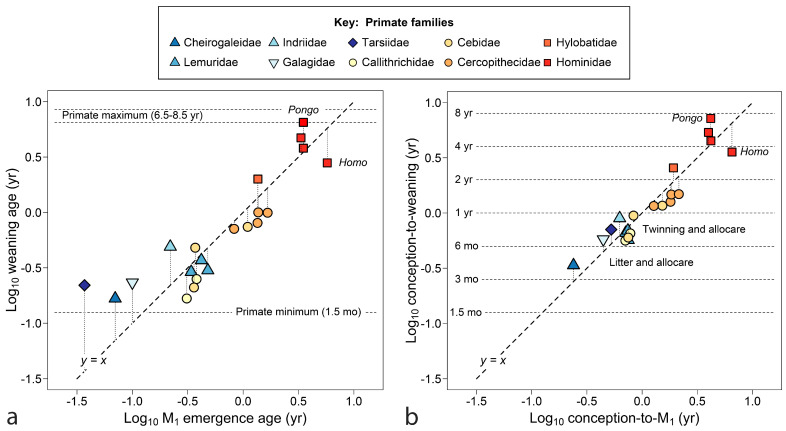
Age of weaning versus age of emergence of M_1_, with age counted from birth (**a**) and conception (**b**) for n = 21 primate species. Residuals (in *y*-direction from the dashed line *y* = *x*) shown for each datum as a fine line. Horizontal dotted lines mark limits for primates (**a**) or doubling of maternal investment (**b**). Twinning, litters and marked allocare occur in species that invest less than one year in their offspring. *Pongo* and *Homo* are diametrically opposed in tooth emergence vs weaning.

The tooth emergence event coincident with weaning turns out to be M_1_, the tooth between dp4 and M_2_ both in emergence timing and spatially in the mouth (Figure 3). Weaning and M_1_ emergence are closest to equivalence over the mid-range (ca. log x = −0.5 to log x = 0.5), suggesting that the two events function well together over a particular time range (0.3 years to 3 years), but are not equally free to vary. Although some primate infants erupt M_1_ earlier than 1.5 months of age, they will not be nursed less than that, a minimum value for the order [27]. At the high end, if humans continued to nurse until M_1_ emerged, near 6 years, the increased interbirth intervals could drive lifetime fertility toward extinction. *Pongo pygmaeus* in Borneo and *P. abelii* in Sumatra are the only primates—indeed the only mammals (see below)—reported with mean interbirth intervals beyond 7 years [98,168], although elephant populations under stress reach similar values [160].

Figure 3b repeats the same scatter counting age from conception. The six doublings shown approach the known range for NVP mammals (33 days to 9.25 years). This perspective reduces residuals from the line for fast-growing primates, with little effect on slow-growing species. Data remain clustered within semi-annual and annual time divisions. The cluster of species with maternal investments of 6–12 months includes species that twin regularly, within Lemuridae, Galagidae and Callitrichidae, groups also known for allocare and paternal care [27,176], and in Callitrichidae, wrapping a new pregnancy with nursing by gestational diapause [177]. The cebid *Aotus* also shows paternal care [27]. The cheirogaleid dwarf lemur, with its strongly seasonal breeding and winter torpor, produces and weans each litter in its active half-year, a feat accomplished with the aid of paternal care. In Figure 3b, humans and orangutans are diametrically opposed, sharing the two largest residuals from *y* = *x*. Humans wean early relative to tooth emergence, whereas orangutans wean late.

**Figure 4:**
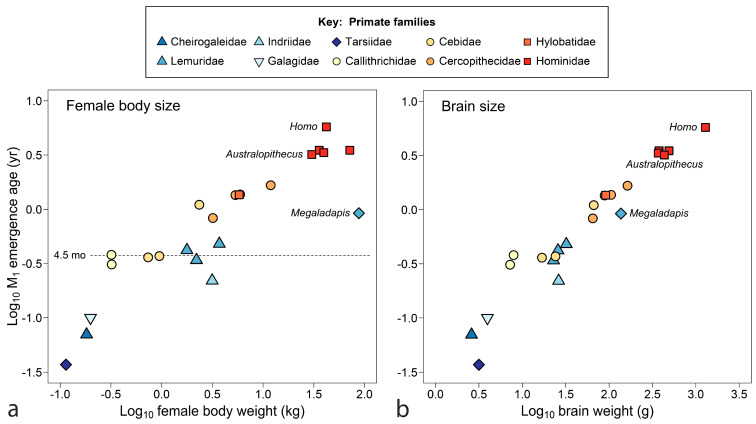
Age of M_1_ emergence on size, measured by female body weight (**a**) and adult brain weight (**b**) for n = 21 living and n = 2 extinct primate species. Stairsteps in *y*-data show that similar ages of tooth emergence span a large range of body weights, especially near 4.5 months (dotted line). Brain size has a tighter linear relationship with tooth emergence, although echoes of the lateral spread in (**a**) remain in (**b**).

The final comparison for primates, Figure 4 relates M_1_ emergence to size, adding data for *Australopithecus africanus* and the large extinct Malagasy lemur *Megladapis edwardsi*. Figure 4a shows tooth emergence as poorly responsive to female body weight. At an emergence age of about 4.5 months (dotted line), weight spans an order of magnitude without increase in age of M_1_ emergence for several South American monkeys (callitrichids and some cebids) and Madagascar lemurs. *Megaladapis*, standing far outside the overall distribution, challenges explanation. Even if adult female body weight (88 kg) is overestimated, weight would have to drop to about 10 kg to merge *Megaladapis* with the primate point cloud.

In Figure 4b, with brain weight as the measure of size, data tighten to something approaching a trend line—indeed, brain weight accounts for both humans and *Megaladapis*. Still, remnants of the flat line at y ≈ 4.5 months can be identified in Figure 4b once seen in Figure 4a. Figure 4 begs other questions: is the persistent emergence time of 4.5 months a grade, clade or niche? Is there another near year 1.0 (zero on the scale of Figure 4)? Although the extinct *Australopithecus* clusters tightly with the living great apes, the extinct *Megaladapis* is so isolated that it is unclear what model should be used to predict M_1_ emergence for another large extinct lemuroid—by grade or sub-group regression? What about a larger tarsiiod? Finally, of course, what is so special about 4.5 months? At least the last question can be addressed below.

### 4.2. All Mammals

#### 4.2.1. The Dentition at Birth

The 47 non-primate species described here range from ‘naked gums’ to all deciduous teeth emerging at birth (Table 2). No cases of permanent teeth emerging at birth were discovered.

The Carnivora are largely altricial, some extremely so, born without teeth showing and held in a nest. Emerging deciduous teeth at birth distinguish the rare precocious species *Crocuta*, *Enhydra* and *Mirounga*. Rodent families may be either precocial or altricial, and here, teeth at birth separate the precocious *Hystrix* and *Thryonomys* from the altricial *Sciurus* and *Myodes*. All the artiodactyls in Table 2 are born with teeth already emerged through the gums at birth, from 6–8 for the *Lagerjunge* suines to 6–22 in the fully precocial species.

A lack of visibly emerged teeth at birth does not translate to overall altriciality for perissodactyls. After a gestation of more than a year, young are born precocial in terms of mobility and thermoregulation but appear to be briefly toothless. In *Equus*, incisors may be just below a membrane at birth and soon cut (rhinos *Ceratotherium* and *Diceros* have no incisors, deciduous or permanent). Tooth development is well along at birth in *Tapirus terrestris* [178], but about a week passes before deciduous incisors erupt [179].

#### 4.2.2. Maternal Investment

Mammals that may reproduce more than once a year are better represented in Table 2, as in *Tupaia*, rodents, smaller carnivores and the tenrec, *Macroscelides.* Mammals that appear just over a boundary may still fit under it in prime years, because averages include non-prime mothers, because pregnancy overlaps with very diminished nursing, or simply because gestation and weaning were estimated independently. For example, *Thyronomys*, the cane rat, totals an investment of 0.503 years, but is said to mate seasonally and to produce two litters per year [107]. Similarly, *Aepyceros*, with an investment of 1.18 years, also reproduces annually in its prime. The category most represented in Table 2, however, is those species that reproduce once per year.

#### 4.2.3. Mapping Weaning onto Tooth Emergence

**Figure 5:**
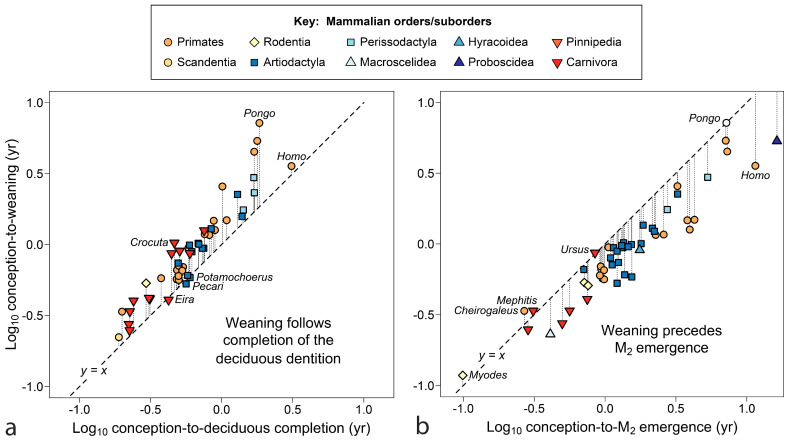
Weaning takes place after completion of the deciduous dentition in 47 out of 50 species in (**a**) and before emergence of M_2_ in 47 out of 52 in (**b**). Age is counted from conception and residuals (in *y*-direction from the line *y* = *x*) are shown as fine lines. Boundary cases: (**a**) suines and *Eira* wean early relative to teeth, (**b**) *Ursus*, *Pongo*, *Mephitis* and *Cheirogaleus* wean late relative to teeth (M_2_ datum for *Pongo* uncertain).

Figure 5 tests the prediction that weaning follows the deciduous dentition and precedes M_2_ as found for primates, counting age from conception.

In Figure 5a, 47 out of 50 species wean after the deciduous dentition is complete, with three borderline cases: the suine artiodactyls *Potomochoerus* and *Pecari* (*Sus* is quite close) and one small carnivore, the tayra (*Eira barbera*). *Crocuta* stands out in the opposite direction, with extended nursing for a carnivore compared to M_1_ emergence (M_2_s have been lost). In Figure 5b, infants are weaned before the emergence of M_2_ in 47 out of 52 cases. With only *Myodes* representing the fastest sector of development, it is difficult to know what is typical in that domain, but the other borderline cases are special: *Mephitis* has no deciduous teeth and the two other cases are well known for their extended maternal investment. If the brown bear, *Ursus arctos*, nurses her cubs over a second winter, nursing would end well past M_2_, as it may be for some species of *Pongo*.

**Figure 6:**
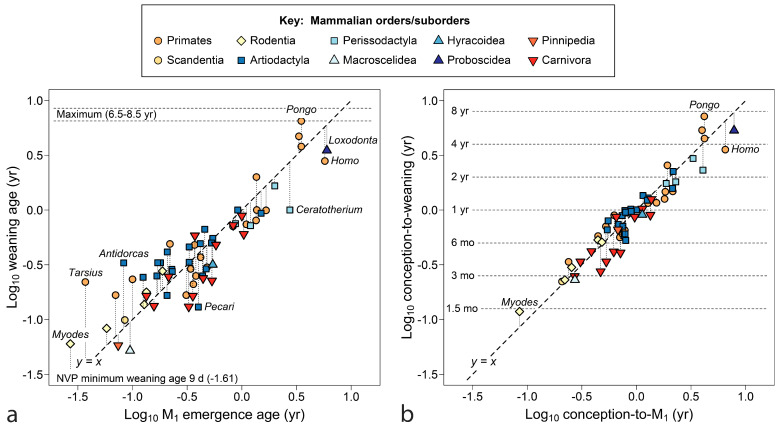
‘Vole to elephant’ plots for the correspondence between age of weaning and M_1_ emergence for 67 mammal species with age counted from birth, (**a**) and from conception, (**b**). Residuals in *y*-direction from the line *y* = *x* are shown as fine lines. Maximum for nonvolant placental mammals dotted in (**a**); doubling of maternal investment dotted in (**b**). Except for apes, slow-growing mammals wean early relative to the M_1_ emergence.

In Figure 6**,** the larger sample of mammals is compared to the line marking equivalent weaning and M_1_ emergence, counting time from birth (a) and conception (b). As before, weaning is most coincident with M_1_ emergence, although fast-developing young are nursed slightly past its emergence and the slowest may wean earlier. In Figure 6a,b, artiodactyls appear more often above the line (fourteen species at or above the line versus six below), reflecting weaning after M_1_ appears, whereas more carnivores lie below it (three at or above vs ten below), more often transiting to adult food sources with only the deciduous dentition (both distributions differ significantly [*p* < 0.05] from random expectation).

In Figure 6b, age is counted from conception, making the *y*-axis reflect maternal investment. Error pulls in for rapidly maturing taxa, but at the slow end, the Hominidae remain divergent from *y = x*. Many taxa, particularly artiodactyls, crowd into the bin of a 6–12-month maternal investment, cut by either axis.

**Figure 7:**
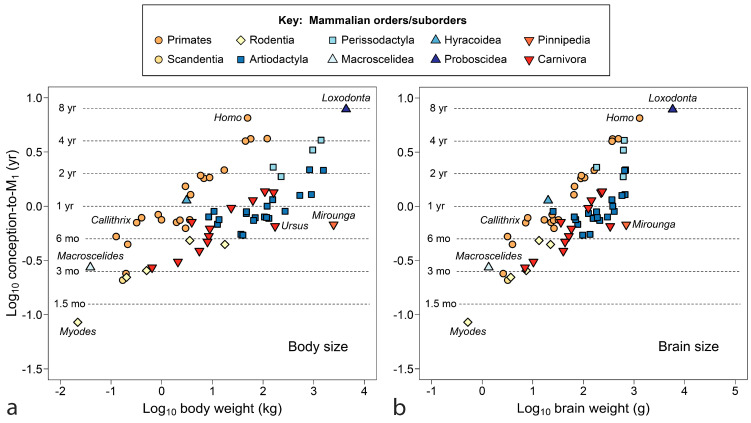
Age of M_1_ emergence counted from conception relative to size, as measured by adult body weight (**a**), and adult brain weight (**b**) for 67 mammal species. Dotted lines represent doublings in time invested to raise young with first permanent molars. Some species that seem extreme in (**a**) are less so in (**b**), but the crowd with investment of ca 11 months remains.

Figure 7 returns to the issue of size, using age at M_1_ emergence counted from conception as a measure of time invested to produce young capable of independent feeding. Within well-represented orders, it could be said that larger species invest more time in their young; however, a flat line in *y*-data interrupts linear trends, where mammals of a truly vast range of size halt short of a one-year investment. The clearest example is *Hyemoschus*, the 10 kg water chevrotain, which invests the same time to produce young capable of independent feeding as does *Alces*, the 270 kg moose (0.896 vs. 0.897 years, Table 2).

With brain size as the independent variable (Figure 7b), distributions tighten and some outliers are explained; however, the density of cases investing slightly less than one year in their offspring remains great.

**Figure 8:**
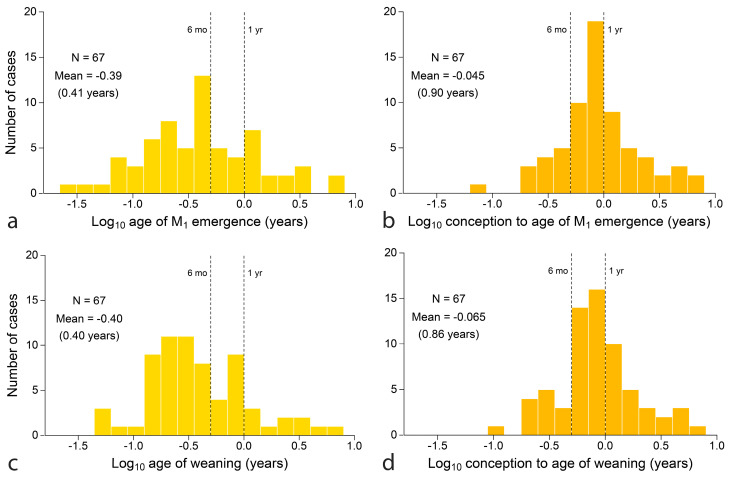
Distributions of estimators of the length of maternal investment, the time used to gestate and raise young to feeding independence: age of M_1_ emergence at top (**a**,**b**) and age of weaning at bottom (**c**,**d**); age is counted from birth at left (**a**,**c**) and from conception at right (**b**,**d**) for 67 species with complete data. When age is counted from conception (**right**), extreme peak values between six months and one year suggest seasonal boundaries have shaped maternal investment. Paired *t*-tests cannot distinguish (**a**) from (**c**), at *p* = 0.78, or (**b**) from (**d**), at *p* = 0.15.

Finally, distributions of key variables can be compared as distributions. Figure 8 compares two measures of the time invested to produce young that feed independently: M_1_ emergence at top (Figure 8a,b) and weaning below (Figure 8c,d). Age is counted from birth (left) and conception (right). At left, both M_1_ emergence and weaning can be studied to some extent in the fossil record, although the mean of the log distributions has no obvious significance. At right, counting age from conception, distributions are peaked with means approaching one year. In common units, both M_1_ emergence and weaning have a geometric mean age of 4.8 months counting from birth and a geometric mean of about 10.8 months counting from conception (Figure 8). Paired t-tests cannot distinguish these two estimators of feeding independence, however, age is counted (*p* = 0.78 and *p* = 0.15, respectively).

## 5. Discussion

The present study attempts to integrate gestation, birth and weaning with tooth emergence for 71 placental mammals, asking how the number of teeth emerged at birth performs as a measure of infant precociality, how weaning maps onto tooth emergence and if a seasonal signal can be found in tooth emergence. Below, after reviewing findings, environmental effects, insights into strategies of slow-growing species (*Ceratotherium*, *Cervus*, *Ursus* and *Pongo*) and approaches to the fossil record are discussed.

*The dentition at birth*: The journey from mammalian embryo to independent feeding juvenile crosses one major threshold—birth. Mammals evolved to differentiate birth status even more widely than hatchling birds [81,82] and generalizations are hard to make beyond ‘mammals do not give birth to adults.’ When we count age from birth, we count from a marker with an enormous range across mammalian development.

Maturity of the dentition, although it represents just one of the organ systems (the digestive) that varies in newborn placentals, is easily quantified, as day of emergence of the first tooth [49], number of teeth at birth (Table 1 and Table 2) or tooth development at birth [33,79]. A gross count of teeth evident at birth has some correspondence with broader categories of birth status within mammalian orders, but imperfectly so. The altricial mammals described here are all born without teeth, but precocious species are more complex. Teeth erupted in the newborn will pick out the precocious rodents and carnivores here; also, fully precocial artiodactyls tend to be born with more teeth than the *Lagerjunge* artiodactyls. Precocious perissodactyls, on the other hand, do not typically cut teeth *in utero*. All primate infants are *Tragerjunge* (parked or carried), but dental precociality at birth varies considerably, advanced in strepsirrhines and the tarsier, but decreasing in ceboids, cercopithecoids, hominoids and hominids—where infancy is long and newborns are toothless.

Dental precociality in mammals ranges beyond that seen in these 71 species. The newborn northern fur seal is erupting M1 and has already shed and replaced anterior teeth [70]. Some South American caviids are born with a full complement of adult teeth [180,181] and at birth, the guinea pigs are already worn from chewing motions in utero [180]. Extreme dental precociality seems to be associated with suppressed deciduous teeth, although it is not the only pathway to that condition [86]. However precocious, all these newborns will be nursed, even if only for a matter of days. Indeed, nursing has been observed to last only four days (range 3–5 days) in a pod of hooded seals (*Cystophora cristata*) born on pack ice off the Labrador coast [182], an even more extreme value than the nine days in Ernest’s compendium [59]. Teeth erupted at birth might reflect a brief milk-only period [6,183], although other explanations are possible, as in the aggressive interactions between litter mates in the spotted hyaena, *Crocuta crocuta* [184].

*Mapping weaning onto tooth emergence*: One clear finding is that ‘milk teeth’ are aptly named because infants are not typically weaned before their final deciduous tooth emerges. The finding holds even in human extractive foragers, the only primate that weans infants who cannot yet feed themselves [163,185] and for young carnivores that may continue to depend on mother’s hunting skills [49]. Seven other mammalian orders follow suit with few exceptions, notably the suines, where typical weaning may slightly precede completion of the deciduous dentition. Although the variability of weaning age is rarely known, data for chimpanzees suggests that even the youngest weaned individual (2–3 years at Gombe, Tanzania) [99] already possesses a complete deciduous dentition, which is finished well before the 2nd birthday in 95% of cases (captive data) [186]. In any case, a bivariate mean locates the centroid around which variation will occur and weaning is not likely to be fully independent of tooth emergence within populations. The co-occurrence of weaning and M_1_ emergence is supported by the 67 mammalian species for which the two variables cannot be shown to differ in direct paired *t*-test (*p* = 0.78).

**Figure 9:**
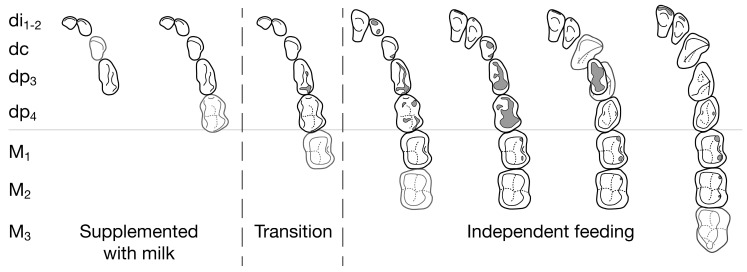
Seven stages of sub-adult life history of mandibular teeth of *Trachypithcus* sp., redrawn and modified from Ingicco et al. [191], mapped against expectations for feeding primates. Teeth in lighter gray are just cutting the gum and shading represents dentin exposure. Findings suggest that three morphological divisions correspond with three stages of feeding: individuals without a full deciduous dentition remain supplemented with milk (infants); the transition to all solid food takes place in the period around the appearance of M_1_ (dashed lines) and individuals with M_2_ emerging are fully independent feeders (juveniles). Permanent I1-P4 replace deciduous predecessors during independent feeding, completing the adult dentition.

Primates are known in sufficient detail that a general model of feeding can be mapped onto dental life history (Figure 9). An incomplete deciduous dentition signifies an infant subsidized by milk. Wear accumulates on deciduous teeth before weaning [187] because learning to eat an adult diet can be an extended process. The transition to independent feeding is predicted to occur with a functional deciduous dentition and a first permanent molar that is emerging or fully erupted, either side of the stage marked off by dashes in Figure 9. Even for the African apes, where nursing can trail out for a year or more after M1s are in full occlusal contact [188,189,190], a functioning di1–M1 is the dentition they take into feeding independence. Independent feeding is predicted to be established for most species by the time M_2_ begins to emerge, which in living primates often occurs after one or more permanent incisors are also in [63].

Although many strepsirrhine primates fit the general model, some are born already at the first division’s dashed line—although not past it [192]; these will wean with a more substantial dental battery [88], although comparisons are not simple to make without more precisely-aged studies.

The primate model appears to extend to other mammals, although the best approach would be to draw a dental life-history map for each mammal order/sub-order. Findings give explicit support for dividing zoological and fossil collections into meaningful age categories by teeth erupted. Perhaps this is no surprise, remembering that Adolph Schultz [193] divided primate life stages by tooth emergence long ago, but it is still remarkable to see data points collected from over 150 completely independent studies firmly support tradition.

*Seasonality and phylogeny in tooth emergence*: Phylogeny, of course, is always in the background. For example, three afrotheres of vastly different size and adaptation, *Loxodonta*, *Procavia* and *Macroscelides* (African elephant, hyrax and tenrec) share an unusual relationship between tooth eruption, skeletal growth and lifespan [194]. Moreover, life histories seem to be quite similar within genera (e.g., *Macaca* sp. in Table 1), see also [26]. Yet, in a seasonal environment, all animals run up against the annual boundary regardless of taxon or size, visible most clearly here in the odd relationship between body size and tooth emergence (Figure 5a and Figure 8a). The odd repetition of M_1_ emergence at 4–5 months in primates is explained by adding gestation length, revealing that the sum of gestation and M_1_ emergence (ca 11 months) is common in other mammalian orders. From chevrotain to moose, squirrel monkey to moose and even marmoset to elephant seal, species of a vast range of size and taxon hold maternal investment (gestation plus lactation or gestation plus age of M_1_ emergence) under one year, presumably because crossing the annual boundary is costly for reproductive fitness. A common eleven-month investment in young presumably means that a one-month recovery is common for mothers between ending lactation and beginning a new pregnancy.

Why so many species crowd the annual border (see Figure 1b, Figure 7a,b and Figure 8b,d) is explained by modeling that shows that seasonal reproduction risks “…time lost by synchronizing innate cycles to environmental cycles,” a cost that is greatest for species with minimum interbirth intervals much less than, or just over, 1.0 year [195] (p. 392). In primates, the most fraught time period of 6–12 months of maternal investment contains species known for twinning and allocare across divergent taxa [176]. Arguably, species within genera and genera within families are kept similar partly because of seasonal reproduction.

It is no great stretch to interpret infant growth and development in the light of maternal investment and reproductive fitness, yet the data merit further study and analytic modeling, e.g., [71,72,196], especially in a manner that includes an avenue to the influence of seasonality [197].

*Environmental effects*: Tooth emergence was observed in captive environments for most taxa in Table 1 and some taxa in Table 2. While evidence shows that tooth emergence timing responds to the level of nutrition, it is far less affected than maturation of bone, linear growth, sexual maturation or body weight [198,199,200,201,202]. Death from debility and starvation has been associated with an average delay of about one standard deviation in some permanent teeth in free-living chimpanzees when compared to a living colony of captives [199], and emergence of the permanent dentition of free-living orangutans remains a current debate [203,204]. There are reasons to expect environmental effects to be largest in teeth forming after deciduous teeth and M1, which are mineralized either in utero or in the early period of maternal nursing. Postnatal nutrition has little effect on the eruption of teeth largely formed in utero, as shown in experimental starvation [202,205,206].

Captive conditions can vary considerably, from mother-nursed to bottle-fed infants. The latter is not common in the studies cited here, although some of the smaller carnivore species were hand-raised (e.g., fox and racoon). For the captive orangutan sample [111], bottle-fed infants could be excluded, which raised the age of completed deciduous dentition from 1.05 to 1.16 years (+6% in developmental age or about 0.5 standard deviations). The Yerkes chimpanzees, a source of M_1_ and M_2_ data, were indeed bottle-fed with preparations commonly used on human infants in the first half of the 20th century [207], but in multiple studies in laboratory and semi-natural conditions, differences in reported emergence ages for chimpanzees remain within sampling variation [186,208].

*Are exceptions revealing? Strategies of slowly growing mammals and the risk of extinction*: The world’s large slow-growing mammals make enormous investments in single offspring, with many months of gestation and years of nursing. This strategy risks extinction if juvenile mortality is not kept very low. Rhinos, for example, are pregnant for 16 months and nurse for one or even two years, varying by population density and forage [209], and weaned calves often remain with their mother until a new infant is born. Rhino calves, however, erupt a major battery of deciduous teeth (dp1-dp4) [119] that, with adequate forage, can support transit to an adult diet months before M_1_ emerges (sometime after two years of age). When conditions are good, this deciduous dental battery can cut many months off weaning time. Keeping these mammals from extinction, as we know, requires controlling mortality from poaching [210], but the large deciduous dental battery keeps young able to take advantage of good conditions.

Investing in present versus future offspring is a central trade-off in life-history [211]. A classic case study of red deer (*Cervus elaphus*) on the island of Rhum [166] measured these very costs, showing some of the factors that influence a hind’s choice to invest more in present offspring by overwintering last spring’s calf, even while it lessens her chance of becoming pregnant the next year. Emerging from the October rut, some pregnant hinds proceed to wean their calf, whereas others continue to nurse into the winter (‘metabolizing for three’). Nonpregnant females also overwinter their calves. Here, molar emergence gives some insight into the growth rate of the young. For this species, averages for age of M_1_ emergence (0.33 yr) and gestation length (0.67 yr) sum to 1.0, evidence that fetal to infant development is closely matched to the annual boundary, if not pressed up against it. Thus, a calf born June 1st cuts its M_1_ in October, the adult rut season. On Rhum, the latest overwinter nursing observed, early July, just precedes M_2_ emergence. Thus, molar emergence seems fitted to life history events. Although they navigate hard seasonal boundaries with young that mature slowly for a cervid, *C. elaphus* remains widely distributed and not widely threatened [212].

The brown bear, *Ursos arctos*, ironically a major predator on *Cervus elaphus*, is another exceptional mother, nursing farther into dental development than other carnivores, often choosing to invest in present, rather than future, offspring. If she weans infants the first summer, they are nearing M_2_ emergence. The sow can extend even more by hibernating with her cubs over second winter, weaning at an average age of 1.15 yrs, and continuing to associate with them into the next year [165]. As molar emergence suggests, these young are juveniles rather than infants.

All the great apes are endangered or critically endangered, with gestations longer than eight months added to years of offspring dependency. Apes do not have a large battery of deciduous teeth and all reports put weaning into the period of emergence of the permanent dentition. Two studies that followed free-living chimpanzees both found that adult feeding patterns coincided with full occlusal contact of upper and lower first permanent molars, some months after these teeth had first emerged [213,214]. All told, raising an offspring to independent feeding requires investments greater than four years for the African apes, a number exceeded by few other living mammals.

The orangutan, all its species critically endangered [215], is reported to maintain increasingly long offspring dependency in recent years [98,216]. No evidence suggests the orangutan infant requires substantially more time than the African apes to grow and develop in captive conditions [111], although orangutans living in a large natural enclosure have been documented to expend less energy relative to their body weight than almost any placental mammal ever measured [217]. Kelley and Schwartz [204] surmise that maturation is delayed in the wild, where Bornean orangutans are now investing—typically—7 ¼ years to gestate and raise an offspring to independent feeding [98], ranging even to ten years in Sumatra [168], while bridging lean periods with nursing, numbers which are highly concerning for conservation. Weaning that occurs some three to five years after young are likely capable of processing adult food bespeaks food sources spread too widely and thinly in space and time for sub-adults [5,98,216]. Reports that orangutan infants in the wild delay the start of solid food for a full year [98], about the age of a completed deciduous dentition, are out of step with other great apes [188,190] and most other mammals. Adding the dentition as a standard reference for growth and development is one way to increase our understanding of the past and present ecology of the orangutan.

*Interpreting the fossil record*: Teeth are unique skeletal structures that do not remodel after they form, preserving a chemical and microstructural record of the physiological conditions of juvenile life. Thus, even adults carry a record of their juvenile period in their permanent teeth and species with ‘ever-growing’ (ever-forming) tusks can record their entire lives [218]. These records can be interpreted almost equally well in living or extinct mammals.

Demonstration that daily and higher-order bandings in mammalian teeth are consistent opened many possibilities for recovering life history in individuals preserved in the fossil record [29,218,219,220,221]. Dental histology has now unlocked information paleontologists thought they would never have—the precise age or season of death—in addition to the timing of dietary and health events [30,34,218,221,222,223,224].

Determining age of tooth ‘emergence’ from a series of juvenile fossils is not in principle different from making the determination in living animals, because gingival emergence is attested by cusp wear and age-of-death determinations can be quite accurate, e.g., [221]; the greater problem is the need to base determinations on a sample, as is now the case for extinct hominins [24]. For example, establishing age at death for a fossil juvenile just beginning to show wear on an erupting M_1_ establishes the age of M_1_ emergence. Indeed, it was just such a 1985 study [29] where a sample of juvenile fossils of the early hominins *Australopithecus* and *Paranthropus* that died while erupting their ‘six-year-molars’ were shown to be close to three years of age at death, a finding that began a revolution in how paleoanthropology viewed Pliocene hominins [225,226,227,228].

Applications of isotopic chemistry to tooth enamel also bloomed after the 1980s, building a library of examples of locating feeding stages preserved in teeth, particularly weaning [229,230,231,232,233].

Newborns are rare in the fossil record [234], but birth initiates profound physiologic changes that are preserved in teeth [16,235] as the ‘neonatal line.’ Its location is likely the best evidence of birth status. It has been proposed that the precocial mammalian orders begin mineralization of the first permanent molar before birth [79], although more evidence on intermediate cases is needed. For example, a neonatal line would be visible only in the apices of a few deciduous teeth in the extremely altricial weasel [236], whereas anthropoids with full-term births record a neonatal line in M_1_, and some strepsirrhines with advanced precociality in M_2_ [79,96]. Data for a growing number of Cenozoic taxa are becoming available [32,79,237]. When and how these differences evolved are questions that could be addressed in the fossil record and compared to a body of theory about why birth status evolves [58,238].

Hard tissues can only preserve a portion of gestation length, so determination of total gestation length seems out of reach for the fossil record. However, Schwartz et al. [239] showed that making inference about gestation length from the partial record preserved in the dentition of precocial primates can make a significant contribution, enough to show that *Megaladapis* and *Archaeolemur* were well over an annual investment in young, a clear vulnerability in Madagascar’s seasonal climate. Rountrey et al. [16] document over a year of gestation for *Mammuthus primigenius* from a juvenile’s tusk. Combining several lines of inquiry [240,241,242], we might estimate gestation length within limits that would allow us to estimate maternal investment in raising offspring to independent feeding in the past—or at least learn the limits of what is knowable.

Mammals today live in the highly seasonal environments prevalent since the beginning of the Oligocene, when the climate switched from “greenhouse earth” to “icehouse earth” [243]. Although most tropical rainforest primates today are seasonal breeders, Eocene climates were not as seasonal as today [244]. Ages of weaning and tooth emergence in the teeth of Paleocene and Eocene mammals might reflect such differences.

For Pleistocene hominins, the early transfer of infant nursing to provisioning with high-quality weaning foods provided by kin is thought to be the key adaptation that allowed humans to keep up our reproductive rate while evolving to slow growth and development to a pace that could support increasingly energetically-demanding brains [57,185,245,246,247,248]. Because both chronological age and food sources are preserved in chemical and structural signatures in teeth [229,230,231,232,233,249,250,251], the evolution of infant provisioning has a fossil record.

In the more recent past, the Pleistocene extinctions left many fewer terrestrial species alive among those that take over one year to produce an independently feeding offspring [252]. Rather than assessing Pleistocene megafauna by body weight alone, reconstructing the age of first molar emergence would take us one step closer to causes of extinction, as in the example for *Megaladapis*, where maternal investment exceeded a one-year investment [33]. Additionally, as the *Megaladapis* record attests (Figure 4), animals from the past can show us what is wrong with models based only on the present.

## 6. Conclusions

The young of toothed mammals cannot reach feeding independence without a functioning dentition. Thus, tooth emergence is one of the “…numerous physiological and behavioral mechanisms link[ing] reproduction and energy metabolism” [41] (p. 1). Tooth eruption has much to recommend it as a life-history descriptor because there are very few measures of physical growth available in life-history studies, despite its importance in theory [253]. Only a few measures of weight at a boundary are commonly studied (neonatal weight, weight at weaning and adult weight). Tooth emergence has the advantages that it adds a measure of skeletal ontogeny, records aspects of birth status and that first permanent molars show a special relationship with weaning. As a life-history variable, perhaps only gestation length has a lower intrinsic variance (e.g., in humans, the coefficient of variation [CV] of gestation days is 4–5% [254] and the CV of tooth emergence is about 10% [26,255]).

Ages of tooth emergence may be reconstructed in the fossil record, expanding cases available to test hypotheses drawn from the ecological present while promising more dynamic inference about life in the past. Its limitations are that mammals that have lost dental homology or lost teeth entirely drop out (e.g., dolphins, baleen whales, modern sirenians, various ant-eating taxa and monotremes) and data remain unavailable for others. Unfortunately, basic reports of tooth emergence that were once standard in the literature of mammalogy and zoological gardens fell out of favor long before mammalian species were described.

Viewing emergence of the teeth of placental mammals in a life-history context leads to the following interpretations, which serve both as present conclusions and hypotheses to be further tested:

1. Broadly, just as seasonal reproduction shapes infant growth, it also shapes timing of emergence of teeth necessary to wean infants onto adult foods. Emergence of the first permanent molar and age of weaning are closely related measures of the time to raise young to independent feeding.
2. The proportion of the dentition formed [79,96] or erupted at birth [26] is one measure of relative infant precociality, although birth status has more than one dimension [81]. Because the proportion of the dentition formed at birth is recorded in hard tissues as the neonatal line, infant precociality has a fossil record.
3. ‘Milk’ teeth are aptly named because the deciduous dentition is typically complete when infants transit fully to an adult diet in nearly all cases examined (47/50). Given a dentition, we can predict a typical feeding stage from teeth erupted, understand the direction of likely exceptions (e.g., highly precocious species) and sort fossil and skeletal collections into meaningful age categories. The emergence of an M_1_ is a reasonable proxy for independent feeding for species in the fossil record.
4. Ages of emergence of the last deciduous and first permanent teeth reveal another layer of meaning when developmental time is counted from conception, because the total time to produce offspring feeding independently comes up against seasonal boundaries that are costly to cross for reproductive fitness. The effect shows strongly for species committed to producing one independently feeding offspring per year.
5. Age of M_1_ emergence is not well explained by future adult body size, although relationships to neonatal and weaning weight [7] remain to be investigated. Brain weight, established much earlier than final adult body weight, appears to be a better proxy for energetics at the boundary of infancy/juvenility, see [256,257]. With adult brain weight as the independent variable, the extremely late emergence of M_1_ in humans is finally explained, although not all clusters of data resolve.
6. The emergence of M_1_ is a useful standard for assessing weaning as early or late across mammals compared to a standard point in growth and development, a comparison that may separate phylogenetic groups, identify dietary guilds and highlight species on the edge of extinction. The orangutan stands as the most divergent mammal in this regard across known species in nine orders, with extremely late weaning in relation to offspring development and tooth emergence.
7. The dental fossil record contains information close to the fundamentals of reproductive effort beyond inferences that can be made from size alone.

## Data Availability

All data used in graphs and analyses appear in Table 1 and Table 2. Worksheets about individual species are available from the author upon request.
